# Idiopathic thrombocytopenic purpura in a patient with *situs inversus totalis*: case report and literature review

**DOI:** 10.31744/einstein_journal/2020RC5111

**Published:** 2019-12-20

**Authors:** Carolina Rodrigues Dal Bo, Beatriz Piovesana Devito, Leticia Piovesana Devito, Gabriella Paes del Papa, Nelson Hamerschlak

**Affiliations:** 1 Faculdade Israelita de Ciências da Saúde Albert Einstein, Hospital Israelita Albert Einstein, São Paulo, SP, Brazil.; 2 Hospital Israelita Albert Einstein, São Paulo, SP, Brazil.

**Keywords:** *Situs inversus*, Purpura, thrombocytopenic, Platelet count, Exome, Hematologic diseases

## Abstract

*Situs inversus totalis* is a rare recessive autosomal congenital abnormality in which the mediastinal and abdominal organs are in a mirrored position when compared to the usual topography. The literature reports some cases of *situs inversus totalis* and concomitant conditions: spinal abnormalities, cardiac malformations and hematological diseases, such as idiopathic thrombocytopenic purpura, which is an autoimmune disease that causes thrombocytopenia due to platelet destruction or suppression of its production. This article aimed to report the coexistence of *situs inversus totalis* and idiopathic thrombocytopenic purpura.

## INTRODUCTION

*Situs inversus totalis* (SIT) is a rare autosomal recessive congenital abnormality in which all mediastinal and abdominal organs are in a mirrored position relative to their normal topography. It is believed that a defect in the long arm (q) of chromosome 14 is responsible for this organ transposition. As a condition, it is compatible with life, and can be asymptomatic.^(^^1^^)^ The incidence is estimated at 1/8,000 to 1/25,000 in liveborns.^(^^2^^)^ Abnormalities in SIT can be recognized first, by using radiography or ultrasonography, and computed tomography is the preferred test for the definitive diagnosis of SIT.^(^^3^^)^ There are reports describing the concurrence of SIT with a variety of other genetic anomalies identified in complete sequencing of the exome.^(^^4^^)^

Idiopathic thrombocytopenia purpura (ITP) is an autoimmune blood disorder characterized by thrombocytopenia due to the destruction of platelets or suppression of their production, by means of an immune reaction against autoantigens on the membranes of the platelets.^(^^5^^)^ The clinical picture may present as critical situations, with cutaneous and mucosal bleeding and even voluminous hemorrhage, which make the quick diagnosis and therapeutic intervention mandatory.^(^^6^^)^ Its incidence is estimated at 1.6 to 2.7 cases per 100 thousand individuals/year, and prevalence at 9.5 to 23.6 cases per 100 thousand individuals, with predominance of the female sex.^(^^7^^)^

This article aimed to report the case of a patient with coexistence of two rare conditions: *situs inversus totalis* and idiopathic thrombocytopenic purpura.

For the literature review, the PubMed database was searched, covering the period from 1975 to 2017. The Medical Subject Headings (MeSH) keywords and terms used in the search were: “purpura”, “*situs inversus totalis*”, “*situs inversus*” AND “purpura”, “*situs inversus*” AND “scoliosis”, “*situs inversus*” AND “whole exome sequencing”.

## CASE REPORT

A 19-year-old male patient, with SIT accidentally identified on imaging tests, reported episodes of intense headache in the frontal and occipital regions with photopsia and loss of consciousness, genital and gingival bleedings, presence of bright red blood in the feces and urine, and findings, on routine examination, of lymphocytopenia and thrombocytopenia. Other complaints reported were dyspnea at rest with spontaneous improvement, pain, and swelling in the lumbar region. As to past history, the patient reported an umbilical hernia, operated on during childhood, but with a relapse. He was a smoker (120 packs-year) and sedentary.

**Figure 1:**
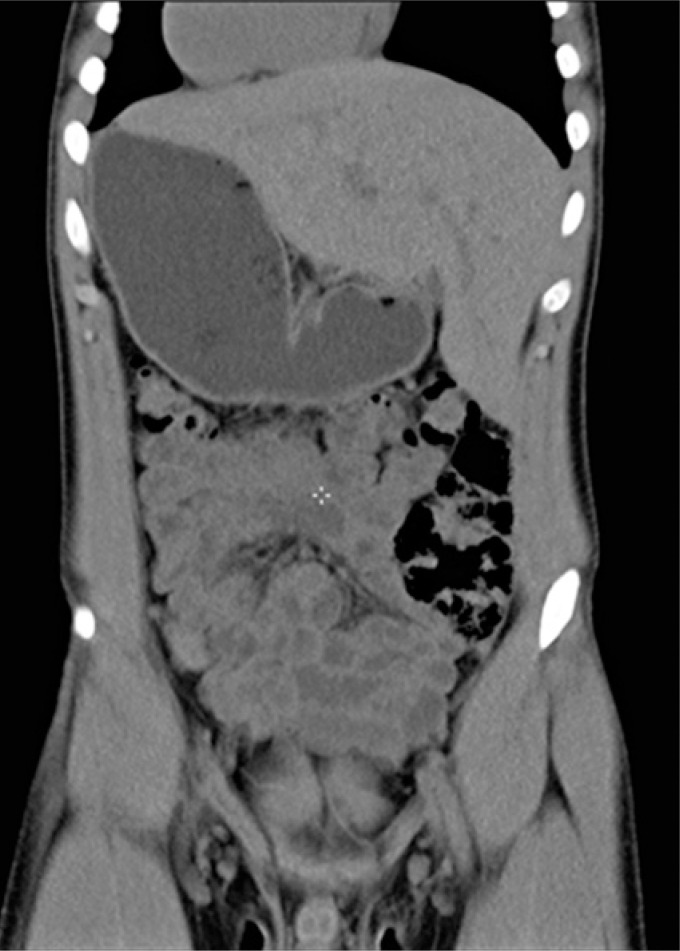
Computed tomography of the total abdomen in coronal view showing abdominal organs in a mirrored position relative to their normal topography

**Figure 2:**
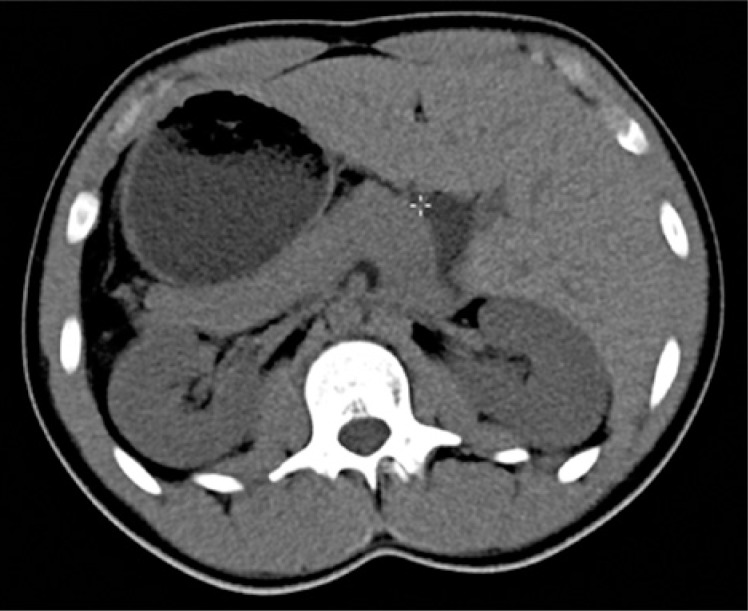
Computed tomography of the total abdomen in cross-sectional view showing abdominal organs in a mirrored position relative to their normal topography

**Figure 3:**
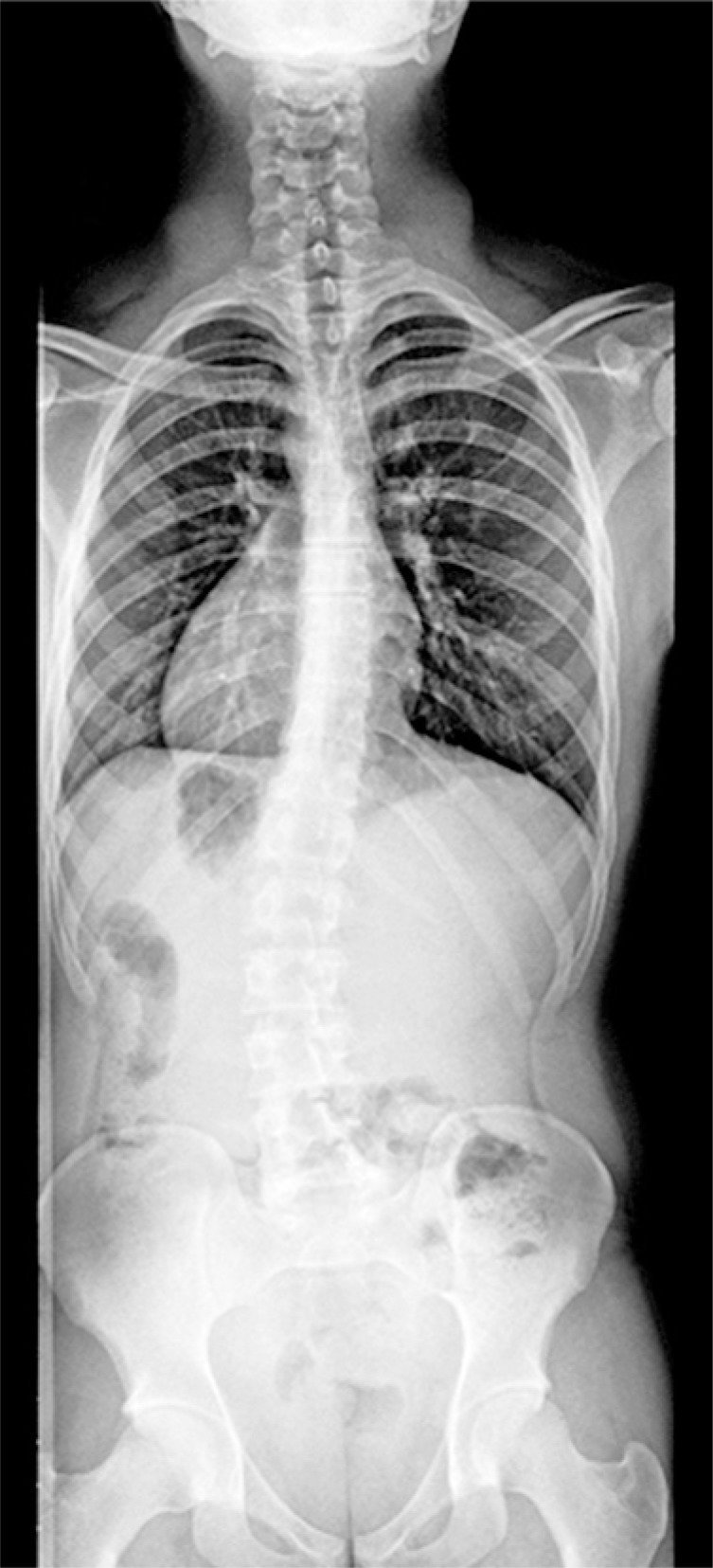
Panoramic radiograph of the total spine showing thoracolumbar sigmoid scoliosis and dextrocardia

**Figure 4:**
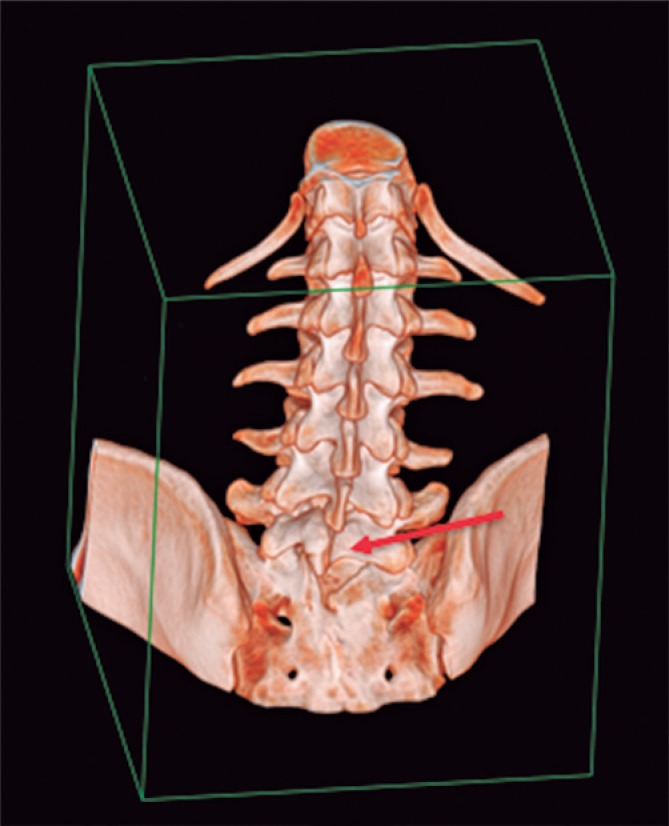
Three-dimension reconstruction showing morphological alteration of the left interapophyseal joint in L5/S1 (red arrow pointing to incomplete fusion of the posterior arch of L5)

A complete blood count (CBC) and imaging tests were performed for investigation. With the results of serial CBC showing thrombocytopenia (minimum value of 64,000/*μ*L), associated with the patient's clinical picture, the clinical and laboratorial diagnosis was made of idiopathic thrombocytopenia purpura. The total abdomen computed tomography (CT) (Figures 1 and 2) showed inversion in the position of abdominal and thoracic structures, confirming SIT. Magnetic resonance of the lumbosacral spine showed lumber scoliosis with convexity to the right in decubitus, a congenital deformity in the posterior arch of L5 and S1 (thinning and deformity), and edema of the spinous ligament of L4/L5. On the radiograph of the total spine (Figure 3), sigmoid thoracolumbar scoliosis with a thoracic component to the left, and a lumbar component to the right, interapophysiary morphologic modification to the left in L5/S1, and signs of incomplete fusion of the posterior arch in L5 were noted. On the CT of the lumbosacral spine, the report showed lumbar scoliosis with convexity to the right in decubitus, and congenital deformity of the posterior arch of L5 and S1 to the left with lysis of the isthmus of L5 (Figure 4).

In conducting the case, when serial CBC were run posteriorly, the patient presented with a variation in the platelet count (64,000/*μ*L up to normality range values). Since it was a case of mild thrombocytopenia tending towards a benign course, watchful waiting was the management chosen, with monthly visits with the hematologist, as well as a monthly CBC with platelet count.

Seeking genetic modifications of SIT, complete sequencing of the exome was performed. No sequence modification variant was sufficient for a molecular diagnosis of SIT was detected. Additionally, two genetic variants were identified: the variant c.580G>A (p. Glu194Lys), in gene FAS in heterozygosis, and the variant c.123_124insCGCGAACGCCAGGCTCGCCGCC p.(Ala42Argfs*31), in gene ADAMTS2, in heterozygosis.

## DISCUSSION

In literature, we found four other cases of ITP^(^^1^^–^^3^^,^^8^^)^ concomitant with SIT. Therefore, this report demonstrates a rare association: the coexistence of SIT, ITP, and spinal anomalies. The causality and the mechanisms of this association are unknown, and subsequent studies should be carried out.

There are studies about the complete sequencing of the exome in patients with primary ciliary dyskinesia and *situs inversus*,^(^^4^^,^^9^^)^ but in literature, we found no investigations that related this genetic study in patients exclusively with SIT. This test has a 30 to 38% rate of diagnostic conclusion. No previously described mutation associated with SIT was found. Some reasons for the negative/inconclusive cases include a pathogenic variant in simple heterozygosis in a recessive gene, absence of identification of variants in genes associated to the phenotype/disease, variants of uncertain significance detected in genes associated with the phenotype/disease, a variant detected that could be deleterious in a gene that currently is not associated with disease in humans.

As to the genetic variants found, the pathological variants in heterozygosis in gene FAS are associated with the autoimmune lymphoproliferative syndrome (ALPS) type IA, of autosomal inheritance, dominant, that occurs with non-malignant lymphadenopathy, hepatoesplenomegaly, autoimmune cytopenias (thrombocytopenia, hemolytic anemia, and neutropenia), which could coincide with ITP and be an autoimmune manifestation of ALPS. Nevertheless, the genetic variant of the FAS gene identified in the patient's exome has an uncertain significance, that is, one cannot say, based on current data, if it is a benign or malignant mutation, considering that it has already been indexed in healthy control patients, and in patients with clinical condition compatible with the mutation. Therefore, further studies are warranted about the human genome in order to reclassify this variant as a cause, or not, of disease.

Pathogenic variants in compound heterozygosis or homozygosis in gene ADAMTS2 are associated with the Ehlers-Danlos syndrome, dermatosparaxis type, of recessive autosomal inheritance, characterized by delay in motor development, low stature, short limbs, capillary weakness, umbilical/inguinal hernia, and gingival bleeding. Since the disease associated with this gene is recessive and only a variant in heterozygosis was detected, this result is interpreted only as the status of the carrier, and not as an affected carrier. However, since exome sequencing has limitations, it is uncertain if the patient has compound heterogeneity, in which it is not possible to rule out pathogenicity. Thus, the umbilical hernia and the gingival bleeding could have been caused by this syndrome.
