# Supplementary material for: Idiopathic thrombocytopenic purpura in a patient with situs inversus totalis: case report and literature review
Source: Einstein (Sao Paulo). 2019 Dec 20;18:eRC5111. doi: 10.31744/einstein_journal/2020RC5111 (PMC6924875; doi:10.31744/einstein_journal/2020RC5111)
Supplement: Translation

# Translation: Púrpura trombocitopênica idiopática em paciente com *situs inversus totalis:* relato de caso e revisão da literatura

**Authors:** Carolina Rodrigues Dal Bo, Beatriz Piovesana Devito, Leticia Piovesana Devito, Gabriella Paes del Papa, Nelson Hamerschlak

**Affiliations:** 1 Faculdade Israelita de Ciências da Saúde Albert Einstein, Hospital Israelita Albert Einstein, São Paulo, SP, Brasil.; 2 Hospital Israelita Albert Einstein, São Paulo, SP, Brasil.

**Keywords:** *Situs inversus*, Púrpura trombocitopênica, Contagem de plaquetas, Exoma, Doenças hematológicas

## Abstract

*Situs inversus totalis* é uma anormalidade congênita autossômica recessiva rara em que os órgãos mediastinais e abdominais encontram-se em posição espelhada em relação à topografia habitual. A literatura relata alguns casos de concomitância do *situs inversus totalis* com outras condições: anomalias espinhais, malformações cardíacas e doenças hematológicas, como púrpura trombocitopênica idiopática, que é uma doença autoimune com plaquetopenia, devido à destruição dos trombócitos ou supressão da sua produção. Esse artigo teve o objetivo de relatar coexistência de *situs inversus totalis* e púrpura trombocitopênica idiopática.

## INTRODUÇÃO

*Situs inversus totalis* (SIT) é uma anormalidade congênita autossômica recessiva rara em que todos os órgãos mediastinais e abdominais encontram-se em posição espelhada em relação à topografia habitual. Acredita-se que um defeito no braço longo (q) do cromossomo 14 é responsável por essa transposição dos órgãos. É uma condição compatível com a vida, que pode ser assintomática.^(^^1^^)^ A incidência é estimada em 1/8.000 a 1/25.000 em nascidos vivos.^(^^2^^)^ Anormalidades no SIT podem ser reconhecidas, primeiro, usando radiografia ou ultrassonografia, sendo a tomografia computadorizada o exame preferido para o diagnóstico definitivo de SIT.^(^^3^^)^ Há relatos já existentes que expõem a concomitância do SIT com uma diversidade de outras anomalias genéticas identificadas no sequenciamento completo do exoma.^(^^4^^)^

Púrpura trombocitopênica idiopática (PTI) é um distúrbio hematológico autoimune caracterizado por plaquetopenia devido à destruição dos trombócitos ou supressão de sua produção, por meio de uma reação imune contra autoantígenos na membrana das plaquetas.^(^^5^^)^ O quadro clínico pode apresentar-se como situações críticas, com sangramento cutâneo e mucoso até hemorragias volumosas, o que torna o diagnóstico rápido e a intervenção terapêutica obrigatórios.^(^^6^^)^ Estima-se que sua incidência seja de 1,6 a 2,7 casos por 100 mil pessoas/ano, e a prevalência de 9,5 a 23,6 casos por 100 mil pessoas, com predominância no sexo feminino.^(^^7^^)^

O objetivo é relatar caso de paciente com coexistência de duas condições raras: *situs inversus totalis* e púrpura trombocitopênica idiopática.

Para revisão bibliográfica do tema, foi consultada a base de dados PubMed, compreendendo o período de 1975 a 2017. Os descritores e termos *Medical Subject Headings* (MeSH) utilizados na busca foram: *“purpura”*, “*situs inversus totalis*”, “*situs inversus*” *AND* “*purpura”, “situs inversus*” *AND* “*scoliosis*”, “*situs inversus*” *AND* “*whole exome sequencing”*.

## RELATO DE CASO

Paciente do sexo masculino, 19 anos, com SIT identificado acidentalmente em exames de imagem, relatou quadros de cefaleia intensa na região frontal e occipital com fotopsia e perda dos sentidos, sangramento genital e gengival, presença de sangue vivo nas fezes e urina, e achados de exame de rotina de linfopenia e plaquetopenia. Outras queixas referidas foram dispneia em repouso com melhora espontânea, dor e inchaço na região lombar. Como antecedente pessoal, relatou hérnia umbilical, operada na infância, mas com recidiva. Era tabagista (120 maços-ano) e sedentário.

Como exames complementares, na investigação, foram realizados hemograma e exames de imagem. Com os resultados de hemogramas seriados demonstrando plaquetopenia (valor mínimo de 64.000/*μ*L), associados ao quadro clínico do paciente, foi feito o diagnóstico de púrpura trombocitopênica idiopática, o qual é clínico e laboratorial. A tomografia computadorizada (TC) de abdome total (Figuras 1 e 2) evidenciou inversão da posição das estruturas abdominais e torácicas, confirmando SIT. A ressonância magnética de coluna lombossacral mostrou escoliose lombar de convexidade para direita no decúbito, deformidade congênita no arco posterior de L5 e S1 (afilamento e deformidade), e edema do ligamento espinhoso de L4/L5. Na radiografia de coluna total (Figura 3), foi evidenciada escoliose toracolombar sigmoide com componente torácico à esquerda e lombar à direita, alteração morfológica da interapofisária esquerda em L5/S1, e sinais de fusão incompleta do arco posterior de L5. Na TC de coluna lombossacral, o laudo mostrou escoliose lombar de convexidade para direita no decúbito e deformidade congênita do arco posterior de L5 e S1 à esquerda, com lise do istmo de L5 (Figura 4).

**Figura 1:** Tomografia computadorizada de abdome total em corte coronal mostrando os órgãos abdominais em posição espelhada em relação à topografia habitual

**Figura 2:** Tomografia computadorizada de abdome total em corte transversal evidenciando os órgãos abdominais em posição espelhada em relação à topografia habitual

**Figura 3:** Radiografia panorâmica de coluna total evidenciando escoliose toracolombar sigmoide e dextrocardia

**Figura 4:** Reconstrução tridimensional evidenciando alteração morfológica da interapofisária esquerda em L5/S1 (seta vermelha aponta fusão incompleta do arco posterior de L5)

Na condução do caso, ao realizar hemogramas seriados posteriormente, o paciente apresentava variação do nível das plaquetas (64.000/*μ*L até os valores da faixa de normalidade). Por ser um caso de plaquetopenia leve que tendia a um curso benigno, optou-se por conduta expectante, com consultas mensais com o médico hematologista, bem como realização mensal de hemograma com contagem de plaquetas.

Em busca de alterações genéticas de SIT, foi realizado sequenciamento completo do exoma. Nenhuma variante de alteração de sequência que seja suficiente para um diagnóstico molecular de SIT foi detectada. Adicionalmente, duas variantes genéticas foram identificadas: a variante c.580G>A (p. Glu194Lys) no gene FAS em heterozigose, e a variante c.123_124insCGCGAACGCCAGGCTCGCCGCC p.(Ala42Argfs*31) no gene ADAMTS2 foi em heterozigose.

## DISCUSSÃO

Encontramos na literatura outros quatro casos de PTI^(^^1^^–^^3^^,^^8^^)^ concomitante ao SIT. Portanto, este relato de caso demonstra associação rara: coexistência de SIT, PTI e anomalias espinhais. A causalidade e os mecanismos destas associações são desconhecidos, e devem ser realizados estudos subsequentes.

Existem estudos sobre sequenciamento completo do exoma em pacientes com discinesia ciliar primária e *situs inversus*,^(^^4^^,^^9^^)^ mas não encontramos na literatura pesquisas que relacionem esse estudo genético em pacientes exclusivamente com SIT. Esse exame possui taxa de conclusão diagnóstica de 30 a 38%. Não foi encontrada mutação associada à SIT já descrita. Algumas razões para os casos negativos/inconclusivos incluem: variante patogênica em heterozigose simples em gene recessivo, ausência de identificação de variantes em genes associados ao fenótipo/doença, variantes de significado incerto detectadas em genes associados ao fenótipo/doença, variante detectada que pode ser deletéria em gene que não está associado ao momento com doença em humanos.

Em relação às variantes genéticas encontradas, as variantes patológicas em heterozigose no gene FAS estão associadas à síndrome linfoproliferativa autoimune (ALPS - *autoimmune lymphoproliferative syndrome*) tipo IA, de herança autossômica, dominante, que cursa com linfadenopatia não maligna, hepatoesplenomegalia, citopenias autoimunes (trombocitopenia, anemia hemolítica e neutropenia), o que poderia coincidir o PTI sendo manifestação autoimune do ALPS. Porém, a variante genética do gene FAS identificada no exoma do paciente possui significado incerto, isto é, não se pode dizer, com base nos dados atuais, se é uma mutação benigna ou maligna, dado que ela já foi catalogada em pacientes controles saudáveis, e em pacientes com patologia clínica compatível com a mutação. Assim, são necessários mais estudos sobre o genoma humano para reclassificar essa variante como causadora ou não de doença.

Variantes patogênicas em heterozigose composta ou homozigose no gene ADAMTS2 estão associadas à síndrome Ehlers-Danlos, tipo dermatoparaxis, de herança autossômica recessiva, caracterizada por atraso do desenvolvimento motor, baixa estatura, membros curtos, fragilidade capilar, hérnia umbilical/inguinal, sangramento gengival. Como a doença associada a esse gene é recessiva e somente uma variante em heterozigose foi detectada, este resultado é interpretado somente como *status* de portador, e não de afetado. Porém, como o sequenciamento do exoma possui limitações, é incerto se o paciente possui heterogeneidade composta, não sendo possível descartar patogenicidade. Assim, a hérnia umbilical e o sangramento gengival podem ter sido causada por essa síndrome.
